# Elevated Total Homocysteine in All Participants and Plasma Vitamin B12 Concentrations in Women Are Associated With All-Cause and Cardiovascular Mortality in the Very Old: The Newcastle 85+ Study

**DOI:** 10.1093/gerona/gly035

**Published:** 2018-02-24

**Authors:** Nuno Mendonça, Carol Jagger, Antoneta Granic, Carmen Martin-Ruiz, John C Mathers, Chris J Seal, Tom R Hill

**Affiliations:** 1 Institute of Cellular Medicine, Newcastle upon Tyne, UK; 2 Newcastle University Institute for Ageing, Newcastle University, Newcastle upon Tyne, UK; 3 Human Nutrition Research Centre, Newcastle University, Newcastle upon Tyne, UK; 4 Institute of Health and Society, Newcastle University, Newcastle upon Tyne, UK; 5 AGE Research Group, Institute of Neuroscience, Newcastle University, Newcastle upon Tyne, UK; 6 NIHR Newcastle Biomedical Research Centre, Newcastle University and Newcastle upon Tyne NHS Foundation Trust, UK

**Keywords:** “Aged, 80 and over”, One carbon metabolism, Folate

## Abstract

**Background:**

Folate and vitamin B12 are keys to the correct functioning of one-carbon (1-C) metabolism. The current evidence on associations between 1-C metabolism biomarkers and mortality is inconclusive and generally based on younger or institutionalized populations. This study aimed to determine the associations between biomarkers of 1-C metabolism and all-cause and cardiovascular (CVD) mortality in the very old.

**Methods:**

The Newcastle 85+ Study is a prospective longitudinal study of participants aged 85 at recruitment living in Northeast England. Baseline red blood cell folate (RBC folate), plasma vitamin B12, and total homocysteine (tHcy) concentrations were available for 752–766 participants. Associations between biomarkers of 1-C metabolism and all-cause and CVD mortality for up to 9 years were assessed by Cox proportional hazard models and confirmed by restricted cubic splines.

**Results:**

Participants with higher tHcy concentrations had higher risk of death from any cause (hazard ratio [HR] [×10 μmol/L]: 1.24, 95% confidence interval [CI]: 1.10–1.41) and cardiovascular diseases (HR [×10 μmol/L]: 1.23, 95% CI: 1.04–1.45) than those with lower concentrations; and women with higher plasma vitamin B12 concentrations had increased risk of all-cause and cardiovascular mortality (HR [×100 pmol/L]: 1.10, 95% CI: 1.04–1.16) after adjustment for key sociodemographic, lifestyle, and health confounders.

**Conclusion:**

Higher concentrations of tHcy in all participants and plasma vitamin B12 in women were associated with increased risk of all-cause and CVD mortality in the very old. This confirms findings for tHcy in younger populations but the adverse relationships between elevated plasma vitamin B12 concentrations and mortality in this setting are novel and require further investigation.

Folate and vitamin B12 are essential for normal function of one-carbon (1-C) metabolism. Folate provides 1-C units for de novo biosynthesis of purines and pyrimidines, transmethylation of homocysteine to methionine and interconversions of serine and glycine (1). Elevated levels of homocysteine may promote endothelial dysfunction, atherothrombosis and lipid peroxidation (2). Vitamin B12 acts as a coenzyme for methionine synthase (MS), responsible for the transmethylation of homocysteine to methionine and consequent production of S-adenosylmethionine (SAM) and methylmalonyl-CoA mutase, which converts methylamalonyl-CoA to succinyl-CoA in the fatty acid synthesis pathway (1). SAM is the universal methyl donor for methylation of all cellular macromolecules, including DNA and histones, and therefore plays a critical role in regulation of gene expression (1). Given the key role in 1-C metabolism, it is no surprise that homocysteine, folate, and vitamin B12 have been associated with cardiovascular disease (CVD) and all-cause mortality. A meta-analysis pooling data from 30 randomized controlled trials (RCTs) reported that folic acid supplementation reduced CVD risk by 4% and the risk of stroke by 10% (3). Some have found that high concentrations of vitamin B12 were associated with all-cause mortality (4,5) and cancer (particularly hematological and, smoking and alcohol related cancers) (6,7) while others did not (8–11). It is hypothesized that “very high” vitamin B12 concentrations may be due to (i) increased release of vitamin B12 from storage, (ii) upregulation of haptocorrin and transcobalamin synthesis, (iii) decreased clearance of vitamin B12 from plasma, and/or (iv) diminished binding affinity of vitamin B12 for transporter proteins (all of which may be associated with disease development) but no clear mechanism has been determined (12,13).

The current evidence for links between 1-C metabolism biomarkers and mortality is inconclusive and based on studies in younger or institutionalized populations. This study aimed to examine associations between red blood cell folate (RBC folate), plasma vitamin B12, and total homocysteine (tHcy) concentrations at baseline, and all-cause and cardiovascular mortality over 9 years in a large sample of very old adults.

## Material and Methods

### The Newcastle 85+ Study

The Newcastle 85+ Study is a prospective longitudinal study of health trajectories and outcomes in the very old which approached all people turning 85 in 2006 (born in 1921), who were registered in participating general practices (NHS), who did not have an end-stage terminal illness and lived in North East England (Supplementary Figure 1). Study details can be found elsewhere (14).

### Biomarkers of 1-C Metabolism

Forty milliliters of blood were drawn from the antecubital vein after an overnight fast and 95% of the samples were sent within 1 hour to the laboratory. tHcy, RBC folate, and plasma vitamin B12 concentrations were determined by immunoassay at baseline (15). tHcy, RBC folate, and plasma vitamin B12 concentrations were available for 766 (74% response rate [766/1042]), 752 (72% response rate [752/1042]), and 753 (72% response rate [752/1042]) participants, respectively, and were categorized into quartiles (Supplementary Methods). tHcy concentrations were also categorized into <11, 11–15, and >15 µmol/L (16) and plasma vitamin B12 into <148, 148–500, and >500 pmol/L. A plasma vitamin B12 concentration <148 pmol/L is commonly used to diagnose deficiency (17) while a concentration >600 pmol/L is used to identify high plasma vitamin B12 concentrations (6,7). Further, an upper limit of 569 pmol/L is used by the department of clinical biochemistry at the Newcastle Royal Victoria Infirmary where samples were analyzed (18). However, because of the low number of participants with plasma vitamin B12 concentrations >600 pmol/L (*n* = 36) and >569 pmol/L (*n* = 41), >500 pmol/L (*n* = 53) was used.

### All-Cause and Cardiovascular Mortality

Information on dates and cause of death (International classification of diseases and related disorders, 10th revision [ICD-10]) were obtained from the Health and Social Care Information Service (now NHS Digital), UK (19). Deaths coded as I00-I99 (Chapter IX of the ICD-10) were used to determine CVD mortality (19). The time to event of interest (all-cause mortality or cardiovascular-specific mortality) was calculated as the time between blood draw (2006–2007) and time of death (censored on April 28, 2015). Mortality follow-up was conducted over 9 years.

### Statistical Analysis

Details for descriptive statistical analysis and normality assessment are shown in Supplementary Methods.

Longitudinal associations between 1-C metabolism biomarkers (continuous, quartiles, and cut-offs) and mortality (all-cause and CVD) were assessed with Kaplan–Meier curves (unadjusted) and Cox proportional hazards models. Briefly, variables used in the models were chosen for their clinical relevance and from univariate analyses while efforts were made not to overfit the data. Model 1 was not adjusted; Model 2 was adjusted for sex and education; Model 3 was additionally adjusted for disease count and Standardized Mini-Mental State Examination score (SMMSE). The tHcy Model 3 was additionally adjusted for renal impairment; Model 4 was also adjusted for body mass index (BMI), physical activity, smoking, alcohol intake; and Model 5 was further adjusted for the other 1-C metabolism biomarkers (eg, the RBC folate model was adjusted for plasma vitamin B12 and tHcy concentrations). Details of the health assessment and disease count are shown in the Supplementary Methods.

To confirm findings and to assess the linearity of relationships, restricted cubic splines were fitted to the final models with five knots at default locations based on quantiles. For example, the quantiles for the tHcy model for all participants were 10, 14, 17, 21, and 32 µmol/L; for the RBC folate model were 370, 639, 868, 1,205, and 2,244 nmol/L; and 102, 178, 232, 307, and 584 pmol/L for plasma vitamin B12. Multicollinearity was checked with tolerance, variation inflation factor (VIF) and eigenvalues. The proportional hazards (PH) assumption for every Cox regression model was tested by visual inspection of plots for log(−log[survival] vs log[time]) and Schoenfeld residuals versus time. All models used met the PH assumption. In the sensitivity analysis, models were further adjusted for: alanine aminotransferase (ALT), alkaline phosphatase, and bilirubin concentrations; or for high sensitivity C-reactive protein (hs-CRP); or folic acid/vitamin B12 supplement users; or housing status; or pyridoxal phosphate (vitamin B6); or total cholesterol. The association between plasma vitamin B12 and all-cause mortality in women by <1 and >1 year of follow-up was also assessed by Cox regression models (unadjusted).

Analyses were carried out in IBM SPSS statistics v22.0 except for inspection of the Schoenfeld residuals and plotting restricted cubic splines where R v3.2.2 (packages “survival” and “rms”) was used. *p* <.05 was used to broadly assess statistical significance and point estimates (with confidence intervals) to assess clinical significance.

## Results

Over the 4,082 person-years of follow-up, 73% (*n* = 564) (13.8 deaths per 100 person-years) of those that had 1-C metabolism biomarkers measured at baseline died. Of these, 53% (*n* = 299) (7.3 deaths per 100 person-years) died from CVD. The percentage of participants who died from CVD during the first year of follow-up was similar (49%) to the whole duration of follow-up. The median survival time was 5.5 (IQR: 2.7–8.0) years.

Data on biomarkers of 1-C metabolism were more likely to be missing from women (11%) than men (5%), from those who lived in institutions (26%) than in the community (7%), from those who had low physical activity (12%) than high physical activity (3%), who were not alcohol drinkers (11%) than those who were (3%) and who had a lower score in the SMMSE. A flowchart of the recruitment and retention profile by 1-C metabolism biomarkers availability over the study period is presented in Supplementary Figure 1.

### Total Homocysteine

There were more men, more renally impaired participants and with lower concentrations of ALT in higher quartiles of tHcy. tHcy quartiles were inversely associated with RBC folate and plasma vitamin B12 concentrations (Table 1).

**Table 1.** Population Characteristics in the Newcastle 85+ Study by Quartiles of Total Homocysteine, RBC Folate, and Plasma Vitamin B12

Total homocysteine (µmol/L)					
	*Q1* (<13.5)	*Q2* (13.5–16.7)	*Q3* (16.7–21.4)	*Q4* (>21.4)	p*
Men (%, *n*)	32 (61)	32 (62)	50 (96)	43 (83)	<.001
Red blood cell folate (nmol/L)	1,272 (896–1,748)	940 (675–1,279)	779 (573–1,084)	680 (477–898)	<.001
Plasma vitamin B12 (pmol/L)	297 (225–430)	230 (185–303)	225 (161–293)	186 (134–262)	<.001
Physical activity (high) (%, *n*)	38 (72)	34 (65)	38 (72)	31 (58)	.02
Renal impairment (%, *n*)	8 (15)	15 (29)	22 (41)	51 (98)	<.001
ALT (U/L)	18 (15–23)	17 (14–21)	16 (13–20)	15 (12–19)	<.001
Red blood cell folate (nmol/L)					
	*Q1* (<612)	*Q2* (612–870)	*Q3* (870–1,280)	*Q4* (>1,280)	p*
Plasma vitamin B12 (pmol/L)	201 (135–280)	216 (159–275)	259 (193–371)	278 (205–391)	<.001
Total homocysteine (µmol/L)	19.9 (16.3–24.6)	18.3 (14.9–22.9)	15.6 (13.0–19.6)	13.8 (11.1–17.4)	<.001
Disease count (mean, SD)	2.0 (1.2)	2.3 (1.3)	2.4 (1.2)	2.4 (1.2)	.01^b^
History of cardiovascular disease (%, *n*)	45 (84)	57 (108)	66 (123)	63 (119)	<.001
Plasma vitamin B12 (pmol/L)					
	*Q1* (<170)	*Q2* (170–232)	*Q3* (232–325)	*Q4* (>325)	p*
Red blood cell folate (nmol/L)	683 (479–992)	838 (605–1,159)	913 (690–1,393)	1058 (745–1,608)	<.001
Total homocysteine (µmol/L)	19.7 (15.9–25.1)	17.3 (14.5–21.8)	15.9 (13.3–19.8)	13.9 (11.1–18.2)	<.001
Disease count (mean, SD)	2.2 (1.3)	2.4 (1.2)	2.2 (1.2)	2.3 (1.2)	.51^b^
ALT (U/L)	16 (13–20)	16 (13–21)	17 (14–21)	17 (14–22)	.03

Notes: Continuous variables are presented as medians and interquartile range unless otherwise stated. History of cardiovascular disease includes cardiac, cerebrovascular, and peripheral vascular diseases. ALT = alanine aminotransferase; Q = quartile; SD = standard deviation.

*Chi-squared test (χ^2^) or Kruskal–Wallis test.

^b^One-way ANOVA.

**Figure 1.**
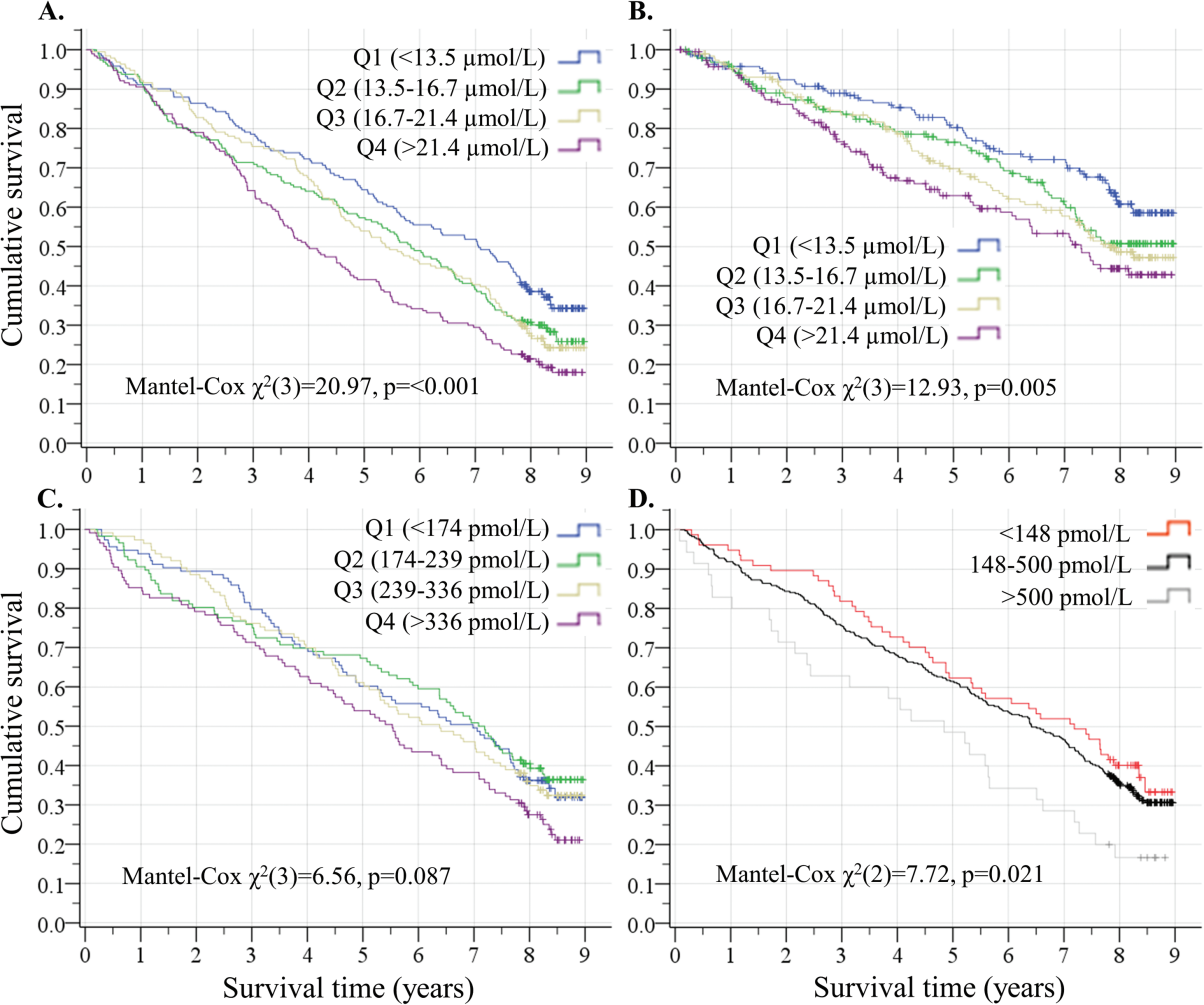
Kaplan–Meier plot of the probability of survival for all-cause (**A**) and cardiovascular mortality (**B**) by total homocysteine quartiles, and all-cause mortality in women by plasma vitamin B12 quartiles (**C**) and reference ranges in women (**D**). Censoring is indicated by crosses.

**Figure 2.**
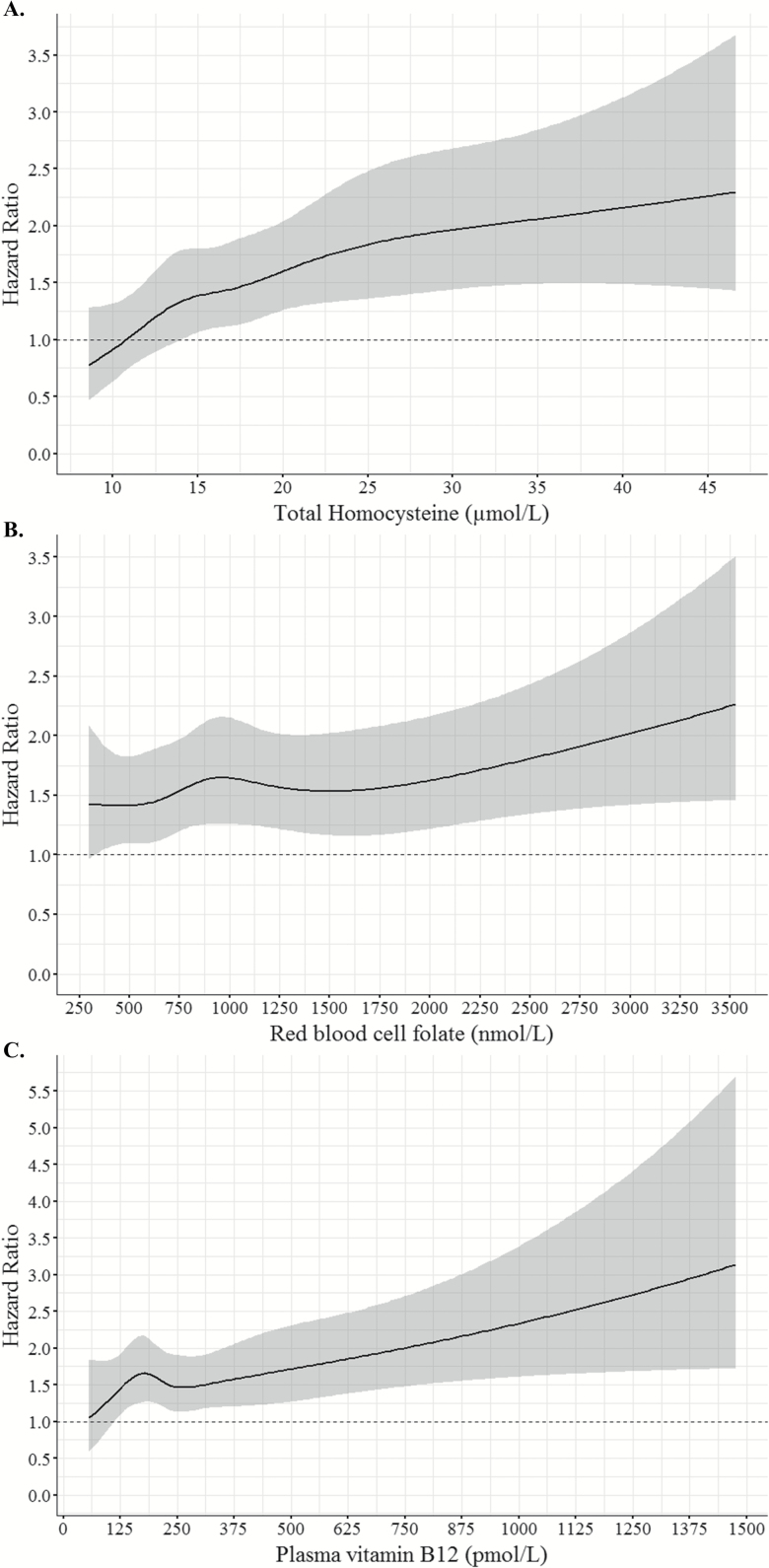
Restricted cubic spline curves of dose–response relationship between total homocysteine (**A**), red blood cell folate (**B**) and plasma vitamin B12 (**C**), and all-cause mortality hazard ratios from the fully-adjusted Cox regression models in Supplementary Table 2 and Table 2.

Survival was longest in Q1 of tHcy (median: 7.1, 95% confidence interval [CI]: 5.9–8.2 years) and lowest in Q4 (median: 4.0, 95% CI: 3.3–4.7) in unadjusted models (Figure 1 and Supplementary Table 1). These differences seemed to emerge after the first year of follow-up (<1 year of follow-up—hazard ratio [HR]: 1.10, 95% CI: 0.87–1.38, *p* = .441 and >1 year—HR: 1.36, 95% CI: 1.21–1.52, *p* < .001; data not shown). For every 10 µmol/L increase in tHcy concentrations the risk of all-cause mortality increased by 24% (HR: 1.24, 95% CI: 1.10–1.41, *p* < .001) in all participants over 9 years after adjustment for socioeconomic and health variables, RBC folate, and plasma vitamin B12 (Supplementary Table 2). Visual inspection of restricted cubic splines of the relationship between tHcy and all-cause mortality confirmed these findings (Figure 2). Results were similar for cardiovascular mortality (Supplementary Tables 2 and 4, Figures 1 and 2, and Supplementary Figures 2 and 3). All relationships between tHcy and mortality were linear (Figure 2 and Supplementary Figure 3). Participants with tHcy concentrations <11 (HR: 0.61, 95% CI: 0.42–0.88, *p* = .008) or 11–15 (HR: 0.78, 95% CI: 0.63–0.97, *p* = 0.028) µmol/L had a lower risk of all-cause mortality than those with >15 µmol/L (Table 3). Results were similar using quartiles of tHcy concentration (Supplementary Table 3).

**Table 2.** Hazard Ratios for All-Cause and Cardiovascular Mortality by Plasma Vitamin B12 (×100 pmol/L) in All Participants, Men, and Women

	All			Men			Women		
	HR	95% CI	*p*	HR	95% CI	*p*	HR	95% CI	*p*
All-cause mortality								
Model 1	1.05	1.01–1.09	.008	1.00	0.94–1.06	.989	1.10	1.05–1.16	<.001
Model 2	1.05	1.01–1.09	.016	1.00	0.94–1.06	.940	1.10	1.05–1.15	<.001
Model 3	1.05	1.01–1.09	.021	0.98	0.92–1.05	.634	1.11	1.06–1.17	<.001
Model 4	1.05	1.01–1.09	.022	1.00	0.93–1.07	.980	1.10	1.04–1.15	<.001
Model 5	1.05	1.01–1.10	.015	1.01	0.94–1.08	.879	1.10	1.04–1.16	<.001
	Cardiovascular mortality								
Model 1	1.05	1.00–1.11	.069	1.01	0.93–1.09	.863	1.09	1.02–1.17	.015
Model 2	1.05	0.99–1.10	.091	1.01	0.93–1.09	.821	1.09	1.02–1.17	.017
Model 3	1.05	1.00–1.11	.073	1.01	0.92–1.09	.915	1.10	1.03–1.18	.007
Model 4	1.06	1.00–1.12	.055	1.01	0.93–1.10	.821	1.09	1.02–1.18	.016
Model 5	1.06	1.00–1.12	.063	1.00	0.91–1.10	.950	1.10	1.02–1.18	.016

Note: Model 1 is not adjusted; Model 2 is adjusted for sex and education; Model 3 is additionally adjusted for disease count and standardized Mini-Mental State Examination score; Model 4 is also adjusted for body mass index, physical activity, smoking, and alcohol intake; Model 5 is further adjusted for total homocysteine and red blood cell folate concentrations. CI = confidence interval; HR = hazard ratio.

### Red Blood Cell Folate

Those in higher quartiles of RBC folate had a slightly higher chronic disease count and more history of CVD than those in lower quartiles (Table 1).

Hazard ratios for all-cause and cardiovascular mortality in RBC folate models in all participants were not significant (Supplementary Tables 2–4, Figure 2, and Supplementary Figures 2 and 3), stratified by sex or length of follow-up (ie, <1 and >1 year; data not shown). All relationships between RBC folate and mortality were linear (Figure 2 and Supplementary Figure 3).

### Plasma Vitamin B12

There were no significant differences in the prevalence of chronic diseases or risk factors between quartiles of plasma vitamin B12 (Table 1). Individuals in different quartiles of plasma vitamin B12 concentration had similar survival rates for all-cause mortality (Supplementary Tables 1 and 3 and Supplementary Figure 2). However, there was a trend for increased hazard ratios in the top quartile of plasma vitamin B12 (>325 pmol/L) compared to the lowest quartile (<170 pmol/L) for cardiovascular mortality (HR: 1.46, 95% CI: 1.00–2.13, *p* = .048) (Supplementary Table 3).

**Figure 3.**
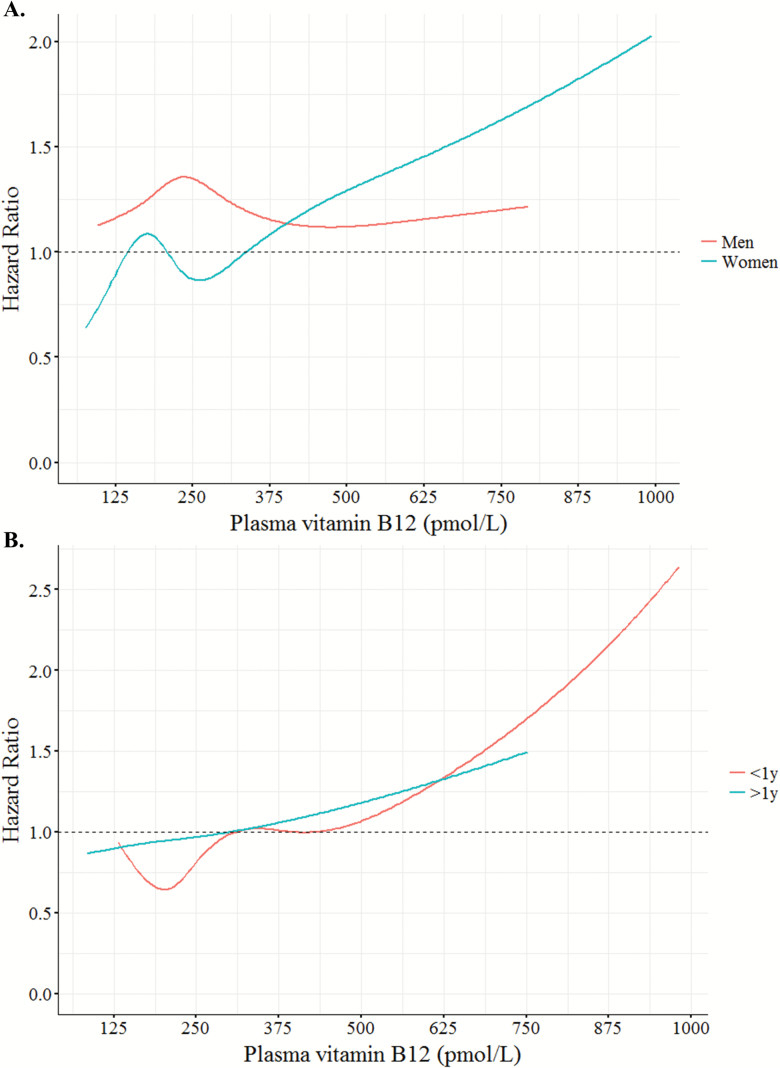
Restricted cubic spline curves of dose–response relationship between plasma vitamin B12 and all-cause mortality hazard ratios for men and women (**A**) from fully-adjusted Cox regression models from Table 2, and by <1 and >1 year of follow-up (**B**) from Supplementary Table 5.

There was a significant sex × plasma vitamin B12 interaction (HR: 1.09, 95% CI: 1.01–1.19, *p* = .036) therefore models were stratified by sex (Table 2). Using plasma vitamin B12 as a continuous variable, for every increase in 100 pmol/L, the risk of all-cause and cardiovascular mortality increased by 10% in women (HR: 1.10, 95% CI: 1.04–1.16, *p* < 0.001 and HR: 1.10, 95% CI: 1.02–1.18, *p* = .016, respectively) but not in men (Table 2). Visual inspection of restricted cubic spline curves confirmed these findings and showed a linear relationship with mortality (except for cardiovascular mortality in women, *p* = .016; Figures 2 and 3). The association between plasma vitamin B12 concentration and mortality in women remained significant if analyses were stratified by <1 and >1 year of follow-up (Figure 3 and Supplementary Table 5).

Seventeen percent (*n* = 128) of participants had plasma vitamin B12 concentrations <148 pmol/L, 76% (*n* = 569) had 148–500 pmol/L and 7.4% (*n* = 56) had >500 pmol/L. Participants with a plasma vitamin B12 >500 pmol/L were at 40% higher risk of all-cause mortality than those with 148–500 pmol/L (HR:1.41, 95% CI: 1.02–1.95, *p* = .039) (Figure 1 and Table 3).

**Table 3.** Hazard Ratios for All-Cause Mortality by Total Homocysteine and Plasma Vitamin B12 Reference Ranges

	Model 1		Model 2		Model 3		Model 4		Model 5	
	HR	95% CI	HR	95% CI	HR	95% CI	HR	95% CI	HR	95% CI
	Total homocysteine (µmol/L)									
<11	0.69	0.50–0.94	0.72	0.52–0.99	0.81	0.59–1.13	0.81	0.58–1.13	0.61	0.42–0.88
11–15	0.69	0.56–0.84	0.73	0.59–0.89	0.82	0.66–1.02	0.85	0.69–1.06	0.78	0.63–0.97
>15	1.00 (reference)		1.00 (reference)		1.00 (reference)		1.00 (reference)		1.00 (reference)	
	Plasma vitamin B12 (pmol/L)									
<148	0.87	0.69–1.10	0.87	0.69–1.10	0.90	0.71–1.13	0.89	0.70–1.13	0.83	0.65–1.08
148–500	1.00 (reference)		1.00 (reference)		1.00 (reference)		1.00 (reference)		1.00 (reference)	
>500	1.31	0.95–1.18	1.28	0.93–1.76	1.30	0.94–1.80	1.39	1.01–1.92	1.41	1.02–1.95

Note: Model 1 is not adjusted; Model 2 is adjusted for sex and education; Model 3 is additionally adjusted for disease count and Mini-Mental State Examination score. The total homocysteine model is additionally adjusted for renal impairment; Model 4 is also adjusted for body mass index, physical activity, smoking, and alcohol intake; Model 5 is further adjusted for the other 1-C metabolism biomarkers. CI = confidence interval; HR = hazard ratio.

## Discussion

During the 4,082 person-years of follow-up, 73% of the participants died and 53% of these deaths were due to CVD. In this longitudinal cohort study of more than 750 participants ≥85 years with 9 years mortality follow-up, men and women with higher baseline tHcy concentrations and women with higher plasma vitamin B12 concentrations (in particular those >500 pmol/L) were at higher risk of death from all-causes and from CVD after adjustment for sociodemographic, lifestyle, and health variables.

Evidence from observational studies confirm our findings that tHcy concentrations are predictive of CVD and/or mortality (2,5,8,11,20–22). Folate donates methyl groups for homocysteine transmethylation to form methionine and, typically, folic acid supplementation lowers homocysteine concentrations by 13%–25% (23,24). Although it is still debatable whether homocysteine is a causal factor or a marker of CVD and mortality (25), others have shown that supplementation with folic acid decreased the risk of CVD, especially stroke (3). There was no significant association between RBC folate concentration and all-cause and cardiovascular mortality—a finding, similar to some studies (26–31) but not others (3,32–34). To the best of our knowledge, the effect of folate on all-cause and cardiovascular mortality has not been investigated in a very old population and the possibility of an age group-specific effect cannot be discounted. The low baseline tHcy concentration or already high folate concentration is frequently pointed out as a limiting factor to detect an effect. However, 57% of the participants in the Newcastle 85+ Study had hyperhomocysteinemia and still no effect was observed (ie, if lowering tHcy is the biological mechanism behind the beneficial effect of folate on mortality).

Our study confirms findings from others where high plasma vitamin B12 was associated with greater all-cause mortality (4,5) and cancer diagnosis/mortality (6,7). Arendt and colleagues (6) identified more than 300,000 people from Danish medical registries not receiving supplemental vitamin B12 and with initial plasma vitamin B12 concentrations >200 pmol/L; categorized them into 200–600, 601–800, and >800 pmol/L and found that cancer incidence increased with higher vitamin B12 concentrations. This effect was more pronounced for hematological, smoking and alcohol-related cancers, and during the first year of follow-up (6). In a further retrospective case–control study of 25,017 patients with cancer diagnosis and plasma vitamin B12 concentrations >200 pmol/L (measured before diagnosis), the same research team reported that patients with a cancer diagnosis who had vitamin B12 concentrations greater than 600 pmol/L had higher risk of mortality than those with concentrations of 200–600 pmol/L (7).

Elevated plasma vitamin B12 concentrations have been found in patients with liver, renal, autoimmune, cancers, and infectious diseases (12). Such diseases may disrupt the uptake of vitamin B12 by the liver, the biggest reservoir of vitamin B12 in the body. Alternatively, increased hepatocyte turnover/damage may lead to greater leakage of liver vitamin B12 (12,35), resulting in higher levels of vitamin B12 in plasma. High plasma vitamin B12 has also been associated with hematological malignancies (12). It is believed that an increase in the number of leukocytes increases haptocorrins’s production and release into the circulation (12). An increase in haptocorrin and subsequent binding of vitamin B12 could lead to reduced holotranscobalamin binding, and hence reduced cellular uptake of vitamin B12 (35). Therefore, it is also possible that, paradoxically, functional deficiency of vitamin B12 occurs concomitantly with elevated plasma vitamin B12 concentrations (35). All of these explanations could account for our findings.

To determine if elevated plasma vitamin B12 was due to liver damage, models were further adjusted for alanine aminotransferase, alkaline phosphatase, and bilirubin; and to determine if it was due to other nonliver related conditions, models were adjusted for hs-CRP. Findings were not significantly different in either of the models. Other sensitivity analyses also did not change the overall conclusions.

The large number of very old adults, the extensive health assessment, the socio-demographic representativeness for the United Kingdom and the rapid processing of blood samples after venepuncture are all considerable strengths of this study. Further, unlike previous literature the majority of the participants lived in the community. However, our study does have limitations. Given that all participants were 85 years at recruitment, an age when mortality rates are high, it is impossible to exclude completely any survival bias due to selective survivability. The use of holotranscobalamin, which measures only the metabolically active fraction of vitamin B12 (while the immunoassay used measures total circulating vitamin B12) would have likely yielded different associations with mortality. Due to the insufficient number of deaths in the first year of follow-up, models stratified by length of follow-up could not be fully adjusted and the hypothesis that (i) 1-C metabolism biomarkers reflect vitamin deficiency and predict long-term mortality or (ii) 1-C biomarkers are markers of disease severity and predict short-term mortality, could not be fully investigated. However, the association between tHcy and mortality seemed to emerge after the first year of follow-up while the association between plasma vitamin B12 and mortality was stronger during the first year of follow-up.

In summary, elevated concentrations of tHcy in men and women and plasma vitamin B12 in women were associated with increased risk of mortality in the very old. This confirms findings for tHcy but the finding of adverse relationships between elevated plasma vitamin B12 concentrations and mortality in mostly community-dwelling very old adults is novel and requires further investigation.

## Supplementary Material

Supplementary data is available at *The Journals of Gerontology, Series A: Biological Sciences and Medical Sciences* online.

Supplemental Files 1

Supplemental Figure 1

Supplemental Figure 2

Supplemental Figure 3

## Funding

This work was supported by a combined grant from the Medical Research Council and Biotechnology and Biological Sciences Research Council (grant number G0500997) and the Dunhill Medical Trust (grant number R124/0509). We also acknowledge the National Health Service for their role in funding and recruitment of participants from general practices.

## Conflict of Interest

The authors report no conflict of interest.
