# Supplementary material for: Elevated Total Homocysteine in All Participants and Plasma Vitamin B12 Concentrations in Women Are Associated With All-Cause and Cardiovascular Mortality in the Very Old: The Newcastle 85+ Study
Source: J Gerontol A Biol Sci Med Sci. 2018 Feb 24;73(9):1258–64. doi: 10.1093/gerona/gly035 (PMC6093381; doi:10.1093/gerona/gly035)
Supplement: Supplemental Files 1 [file gly035_suppl_supplemental_files.docx]

**Elevated total homocysteine in all participants and plasma vitamin B12 concentrations in women are associated with all-cause and cardiovascular mortality in the very old: The Newcastle 85+ Study**

Nuno Mendonça, RD MSc PhD^1-4^; Carol Jagger MSc PhD^2,4^; Antoneta Granic MA PhD^5-6^; Carmen Martin-Ruiz MSc PhD^2^; John C. Mathers PhD^1-3^; Chris J. Seal, PhD^1,3^; Tom R. Hill, PhD^1-3^

^1^ Institute of Cellular Medicine, Newcastle upon Tyne NE2 4HH, UK.

^2^ Newcastle University Institute for Ageing, Newcastle University, Newcastle upon Tyne NE2 4AX, UK.

^3^ Human Nutrition Research Centre, Newcastle University, Newcastle upon Tyne NE2 4HH, UK.

^4^ Institute of Health and Society, Newcastle University, Newcastle upon Tyne NE2 5PL, UK.

^5^ AGE Research Group, Institute of Neuroscience, Newcastle University, Newcastle upon Tyne NE2 4HH, UK.

^6^ NIHR Newcastle Biomedical Research Centre, Newcastle University and Newcastle upon Tyne NHS Foundation Trust, Newcastle upon Tyne NE4 5PL, UK.

**Word count:** 4593 (including tables, figures and references).

**Number of figures:** 3 and 3 supplemental.

**Number of tables:** 3 and 5 supplemental.

**Running title:** 1-C biomarkers and mortality.

**Corresponding author:** Nuno Mendonҫa, nuno.mendonca@newcastle.ac.uk

**Supplemental Methods**

*Biomarkers of 1-C metabolism*

Models were also fit for quartiles of RBC folate - Quartile 1 (Q1):<612, Q2:612-870, Q3:870-1280, Q4:>1280 nmol/L); tHcy - Q1:<13.5, Q2:13.5-16.7, Q3:16.7-21.4, Q4:>21.4 µmol/L; and plasma vitamin B12 - Q1:<170, Q2:170-232, Q3: 232-325, Q4:>325 pmol/L.

*Descriptive statistical analysis and normality assessment*

Normality was assessed graphically with the aid of Q-Q plots and histograms. Normally distributed continuous values are presented as means and standard deviations (SD), and non-Gaussian distributed variables as medians and interquartile ranges (IQR). Categorical data are presented as percentages (with corresponding sample size). Differences between quartiles of 1-C metabolism biomarkers were assessed with chi-squared test (χ2) for categorical variables, one-way ANOVA for continuous parametric and Kruskal-Wallis for non-parametric variables.

*Health assessment and disease count*

Multi-dimensional health questionnaires recorded sex, years of full time education, housing, physical activity, alcohol intake (confirmed by 2x24h multiple pass recalls), supplements use, and smoking status. Information on supplement use was limited to type and brand, therefore folic acid and vitamin B12 supplements were assumed to be taken according to manufacturer’s specifications. Medical records held by the GP were reviewed for diagnosed diabetes mellitus type 1 and 2, hypertension, cardiac disease (heart failure, angina, myocardial infarction, coronary artery bypass graft, coronary angioplasty/stent, pacemaker, atrial flutter/fibrillation), cerebrovascular disease (stroke, transient ischaemic attack, carotid endarterectomy), peripheral vascular disease, arthritis, respiratory disease, and cancer diagnosed within the previous 5 years excluding non-melanoma skin cancer. A disease count was created by scoring these diseases as either present or absent (Kingston *et al*, 2014). The Standardized Mini-Mental State Examination (SMMSE) 30-point scale was used to assess global cognitive function. Weight and height were measured and used to calculate body mass index (BMI). A participant with an estimated glomerular filtration rate < 30ml/minute/1.73m^2^ was considered renally impaired.

## Supplemental Tables

## Supplemental Table 1. Survival time and deaths by quartiles of biomarkers of one-carbon metabolism.

| **Total homocysteine** (µmol/l) | **Q1** (<13.5) | **Q2** (13.5-16.7) | **Q3** (16.7-21.4) | **Q4** (>21.4) |
| --- | --- | --- | --- | --- |
| Survival time (years) | 7.1 (3.3-8.3) | 5.8 (2.5-8.0) | 5.4 (3.2-7.9) | 4.1 (2.4-7.5) |
| Deaths (%, n) | 64 (122) | 72 (138) | 74 (142) | 80 (154) |
| Cardiovascular deaths (%, n) | 33 (62) | 38 (73) | 42 (81) | 43 (82) |
| **Red blood cell folate (**nmol/L) | **Q1** (<612**)** | **Q2** (612-870) | **Q3** (870-1280) | **Q4** (>1280) |
| Survival time (years) | 5.6 (2.9-8.1) | 5.5 (2.5-8.1) | 5.3 (2.9-7.9) | 5.7 (2.6-7.9) |
| Deaths (%, n) | 70 (130) | 70 (133) | 75 (140) | 75 (141) |
| Cardiovascular deaths (%, n) | 34 (64) | 36 (68) | 43 (79) | 42 (80) |
| **Plasma vitamin B12** (pmol/L) | **Q1 (**<170) | **Q2** (170-232) | **Q3** (232-325) | **Q4** (>325) |
| Survival time (years) | 5.6 (3.3-8.2) | 6.1 (2.2-8.0) | 5.3 (2.6-7.9) | 5.2 (2.5-7.9) |
| Deaths (%, n) | 71 (134) | 70 (130) | 75 (140) | 76 (142) |
| Cardiovascular deaths (%, n) | 31 (59) | 40 (74) | 43 (81) | 42 (78) |

Values are medians and interquartile range unless otherwise mentioned. Q, quartile.

**Supplemental Table 2. Hazard ratios for all-cause and cardiovascular mortality by total homocysteine and red blood cell folate in all participants.**

|  | **Total homocysteine** (x10 µmol/L) | | | **Red blood cell folate** (x100 nmol/L) | | |
| --- | --- | --- | --- | --- | --- | --- |
|  | HR | 95% CI | *p* | HR | 95% CI | *p* |
| **All-cause mortality** | | | | | | |
| **Model 1** | 1.35 | 1.21-1.50 | <0.001 | 1.01 | 1.00-1.03 | 0.039 |
| **Model 2** | 1.32 | 1.18-1.47 | <0.001 | 1.02 | 1.00-1.03 | 0.027 |
| **Model 3** | 1.20 | 1.06-1.36 | 0.004 | 1.01 | 0.99-1.03 | 0.078 |
| **Model 4** | 1.13 | 1.01-1.28 | 0.042 | 1.01 | 0.99-1.02 | 0.074 |
| **Model 5** | 1.24 | 1.10-1.41 | 0.001 | 1.01 | 0.99-1.03 | 0.064 |
| **Cardiovascular mortality** | | | | | | |
| **Model 1** | 1.35 | 1.17-1.56 | <0.001 | 1.02 | 1.00-1.04 | 0.025 |
| **Model 2** | 1.32 | 1.14-1.53 | <0.001 | 1.02 | 1.00-1.04 | 0.018 |
| **Model 3** | 1.16 | 0.98-1.36 | 0.089 | 1.02 | 0.99-1.03 | 0.067 |
| **Model 4** | 1.11 | 0.95-1.30 | 0.202 | 1.02 | 0.99-1.03 | 0.074 |
| **Model 5** | 1.23 | 1.04-1.45 | 0.014 | 1.02 | 0.99-1.03 | 0.086 |

Model 1 is not adjusted; Model 2 is adjusted for sex and education; Model 3 is additionally adjusted for disease count and Standardized Mini-Mental State Examination score. The total homocysteine model is additionally adjusted for renal impairment; Model 4 is also adjusted for body mass index, physical activity, smoking and alcohol intake; Model 5 is further adjusted for the other 1-C metabolism biomarkers. CI, confidence interval; HR, hazard ratio.

## Supplemental Table 3. Hazard ratios for all-cause mortality by quartiles of total homocysteine, red blood cell folate and plasma vitamin B12.

|  | **Model 1** | | **Model 2** | | **Model 3** | | **Model 4** | | **Model 5** | |
| --- | --- | --- | --- | --- | --- | --- | --- | --- | --- | --- |
| Quartiles | HR | 95% CI | HR | 95% CI | HR | 95% CI | HR | 95% CI | HR | 95% CI |
|  | **Total Homocysteine** (µmol/L) | | | | | | | | | |
| <13.5 | 1.00 (reference) | | 1.00 (reference) | | 1.00 (reference) | | 1.00 (reference) | | 1.00 (reference) | |
| 13.5-16.7 | 1.31 | 1.01-1.69 | 1.33 | 1.03-1.72 | 1.36 | 1.06-1.76 | 1.54 | 1.19-2.00 | 1.77 | 1.35-2.31 |
| 16.7-21.4 | 1.36 | 1.06-1.76 | 1.25 | 0.97-1.62 | 1.13 | 0.87-1.46 | 1.19 | 0.91-1.54 | 1.40 | 1.07-1.84 |
| >21.4 | 1.82 | 1.42-2.33 | 1.73 | 1.35-2.22 | 1.52 | 1.16-1.99 | 1.52 | 1.16-1.99 | 2.04 | 1.51-2.77 |
|  | **Red Blood Cell Folate** (nmol/L) | | | | | | | | | |
| <612 | 1.00 (reference) | | 1.00 (reference) | | 1.00 (reference) | | 1.00 (reference) | | 1.00 (reference) | |
| 612-868 | 1.03 | 0.81-1.32 | 1.05 | 0.82-1.35 | 1.09 | 0.85-1.40 | 1.04 | 0.81-1.34 | 1.05 | 0.82-1.36 |
| 868-1280 | 1.14 | 0.87-1.42 | 1.15 | 0.90-1.46 | 1.16 | 0.90-1.49 | 1.15 | 0.90-1.48 | 1.19 | 0.92-1.54 |
| >1280 | 1.12 | 0.87-1.42 | 1.14 | 0.89-1.45 | 1.13 | 0.88-1.45 | 1.13 | 0.88-1.45 | 1.16 | 0.90-1.50 |
|  | **Plasma Vitamin B12** (pmol/L) | | | | | | | | | |
| <170 | 1.00 (reference) | | 1.00 (reference) | | 1.00 (reference) | | 1.00 (reference) | | 1.00 (reference) | |
| 170-232 | 1.03 | 0.80-1.31 | 1.04 | 0.81-1.33 | 1.04 | 0.81-1.33 | 0.95 | 0.74-1.22 | 0.98 | 0.76-1.27 |
| 232-325 | 1.14 | 0.90-1.45 | 1.17 | 0.92-1.48 | 1.11 | 0.87-1.41 | 0.99 | 0.77-1.26 | 1.02 | 0.79-1.33 |
| >325 | 1.18 | 0.92-1.50 | 1.20 | 0.94-1.53 | 1.14 | 0.90-1.46 | 1.07 | 0.84-1.37 | 1.08 | 0.83-1.41 |

Model 1 is not adjusted; Model 2 is further adjusted for sex and education; Model 3 is additionally adjusted for disease count and Mini-Mental State Examination score: Model 4 is also adjusted for body mass index, physical activity, smoking and alcohol intake; Model 5 is further adjusted for the other 1-C metabolism biomarkers [e.g. Red blood cell folate model is adjusted for plasma vitamin B12 and total homocysteine (continuous)]. The total homocysteine model is additionally adjusted for renal impairment. CI, confidence interval; HR, hazard ratio.

**Supplemental Table 4. Hazard ratios for cardiovascular mortality by quartiles of total homocysteine, red blood cell folate and plasma vitamin B12.**

|  | **Model 1** | | **Model 2** | | **Model 3** | | **Model 4** | | **Model 5** | |
| --- | --- | --- | --- | --- | --- | --- | --- | --- | --- | --- |
| Quartiles | HR | 95% CI | HR | 95% CI | HR | 95% CI | HR | 95% CI | HR | 95% CI |
|  | **Total Homocysteine** (µmol/L) | | | | | | | | | |
| <13.5 | 1.00 (reference) | | 1.00 (reference) | | 1.00 (reference) | | 1.00 (reference) | | 1.00 (reference) | |
| 13.5-16.7 | 1.34 | 0.94-1.89 | 1.37 | 0.97-1.94 | 1.39 | 0.98-1.97 | 1.53 | 1.07-2.18 | 1.77 | 1.23-2.55 |
| 16.7-21.4 | 1.49 | 1.06-2.09 | 1.36 | 0.96-1.91 | 1.20 | 0.85-1.70 | 1.28 | 0.90-1.82 | 1.54 | 1.07-2.23 |
| >21.4 | 1.84 | 1.31-2.59 | 1.76 | 1.25-2.47 | 1.45 | 1.00-2.10 | 1.47 | 1.01-2.13 | 2.05 | 1.36-3.11 |
|  | **Red Blood Cell Folate** (nmol/L) | | | | | | | | | |
| <612 | 1.00 (reference) | | 1.00 (reference) | | 1.00 (reference) | | 1.00 (reference) | | 1.00 (reference) | |
| 612-868 | 1.04 | 0.74-1.47 | 1.06 | 0.75-1.49 | 1.06 | 0.75-1.50 | 0.99 | 0.70-1.41 | 1.00 | 0.70-1.42 |
| 868-1280 | 1.22 | 0.88-1.70 | 1.26 | 0.90-1.75 | 1.20 | 0.86-1.68 | 1.18 | 0.84-1.65 | 1.21 | 0.85-1.71 |
| >1280 | 1.24 | 0.89-1.72 | 1.26 | 0.90-1.75 | 1.18 | 0.85-1.66 | 1.16 | 0.83-1.63 | 1.18 | 0.83-1.67 |
|  | **Plasma Vitamin B12** (pmol/L) | | | | | | | | | |
| <170 | 1.00 (reference) | | 1.00 (reference) | | 1.00 (reference) | | 1.00 (reference) | | 1.00 (reference) | |
| 170-232 | 1.34 | 0.95-1.90 | 1.37 | 0.97-1.93 | 1.35 | 0.96-1.91 | 1.26 | 0.89-1.79 | 1.33 | 0.93-1.91 |
| 232-325 | 1.49 | 1.06-2.08 | 1.51 | 1.08-2.12 | 1.45 | 1.03-2.03 | 1.32 | 0.94-1.86 | 1.39 | 0.97-2.00 |
| >325 | 1.49 | 0.92-1.50 | 1.53 | 1.09-2.16 | 1.46 | 1.03-2.05 | 1.42 | 1.01-1.86 | 1.46 | 1.00-2.13 |

Model 1 is not adjusted; Model 2 is further adjusted for sex and education; Model 3 is additionally adjusted for disease count and Mini-Mental State examination score: Model 4 is also adjusted for body mass index, physical activity, smoking, alcohol intake; Model 5 is further adjusted for the other 1-C metabolism biomarkers [e.g. Red blood cell folate model is adjusted for plasma vitamin B12 and total homocysteine (continuous)]. The total homocysteine model is additionally adjusted for renal impairment. CI, confidence interval; HR, hazard ratio.

**Supplemental Table 5. Hazard ratios for all-cause mortality by plasma vitamin B12 (x100 pmol/L) in women by <1 and >1 year of follow-up.**

| **Plasma vitamin B12 (x100 pmol/L) Women** | | | | | | |
| --- | --- | --- | --- | --- | --- | --- |
|  | **<1 year** | | | **>1 year** | | |
|  | HR | 95% CI | *p* | HR | 95% CI | *p* |
| **Model 1** | 1.16 | 1.02-1.33 | 0.026 | 1.09 | 1.03-1.15 | 0.002 |

Model 1 is not adjusted. No further adjustment was made due to lack of power. CI, confidence interval; HR, hazard ratio.

## Supplemental Figures

**Supplemental Figure 1.** Flowchart of the recruitment and retention profile of the Newcastle 85+ Study by one-carbon metabolism biomarkers availability and mortality over 9 years.

**Supplemental Figure 2.** Kaplan-Meier plot of the probability of survival by red blood cell folate concentration quartiles for all-cause (**A.**) and cardiovascular mortality (**B.**); and by plasma vitamin B12 quartiles for all-cause (**C.**) and cardiovascular mortality (**D.**). Censoring is indicated by crosses.

Supplemental Figure 3. Restricted cubic spline curves of dose-response relationship between total homocysteine (**A.**), red blood cell folate (**B.**) and plasma vitamin B12 (**C.**), and cardiovascular mortality hazard ratios from the fully-adjusted Cox regression models in **Supplemental Table 2** and **Table 2**.
