# Supplementary figures and images for: Elevated Total Homocysteine in All Participants and Plasma Vitamin B12 Concentrations in Women Are Associated With All-Cause and Cardiovascular Mortality in the Very Old: The Newcastle 85+ Study
Source: J Gerontol A Biol Sci Med Sci. 2018 Feb 24;73(9):1258–64. doi: 10.1093/gerona/gly035 (PMC6093381; doi:10.1093/gerona/gly035)

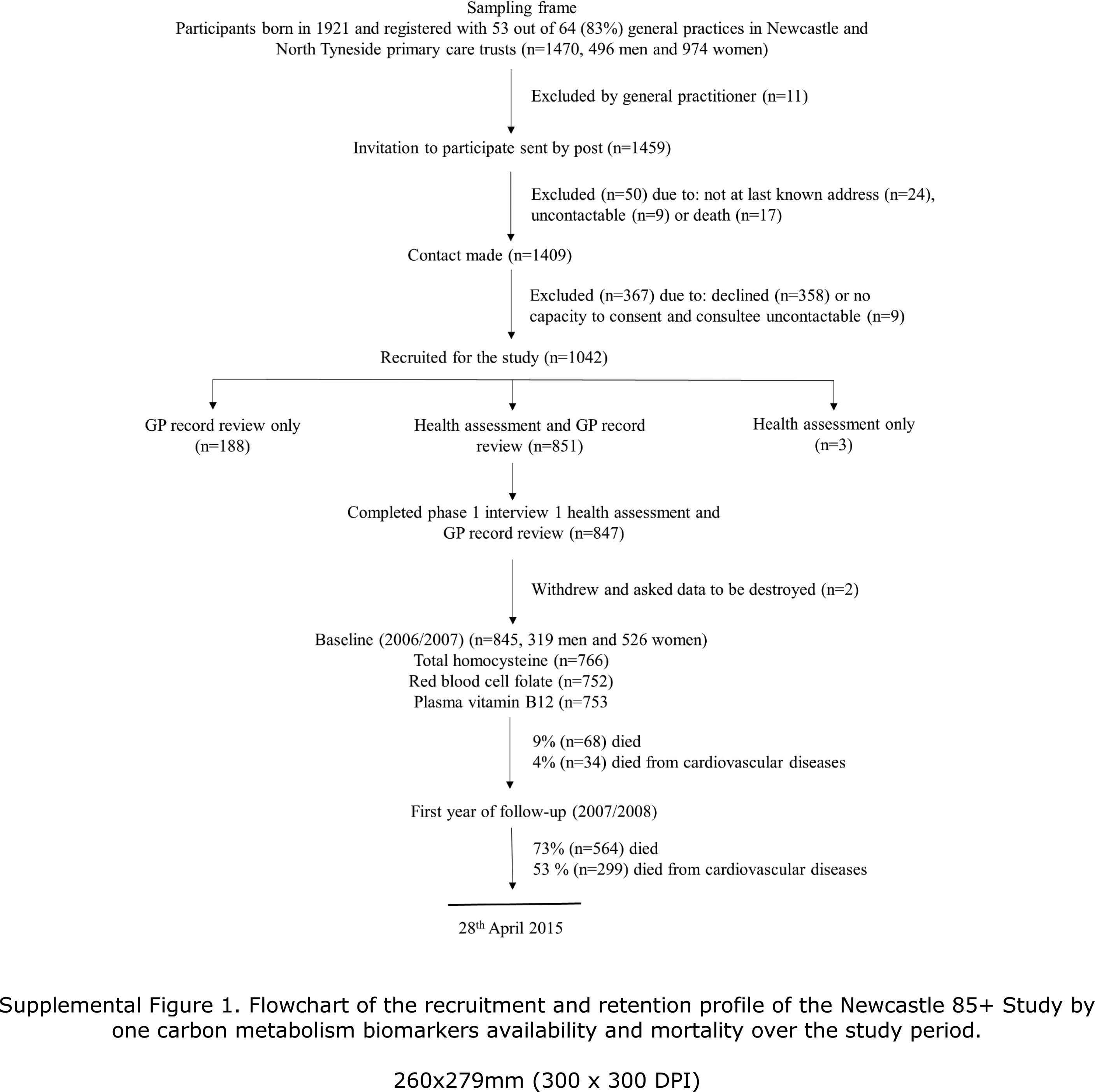

Supplement: Supplemental Figure 1 [file gly035_suppl_supplemental_figure_1.png]

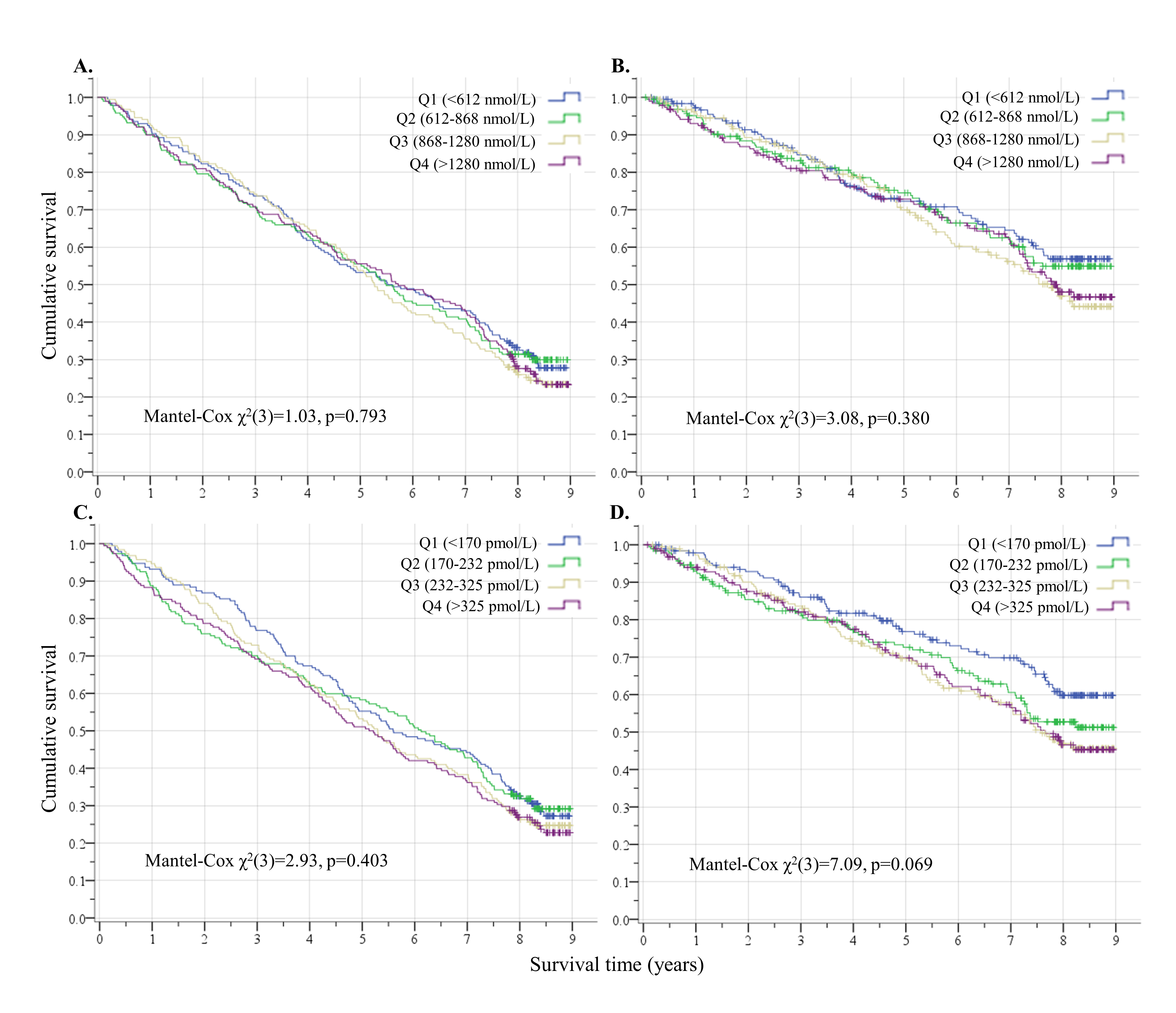

Supplement: Supplemental Figure 2 [file gly035_suppl_supplemental_figure_2.png]

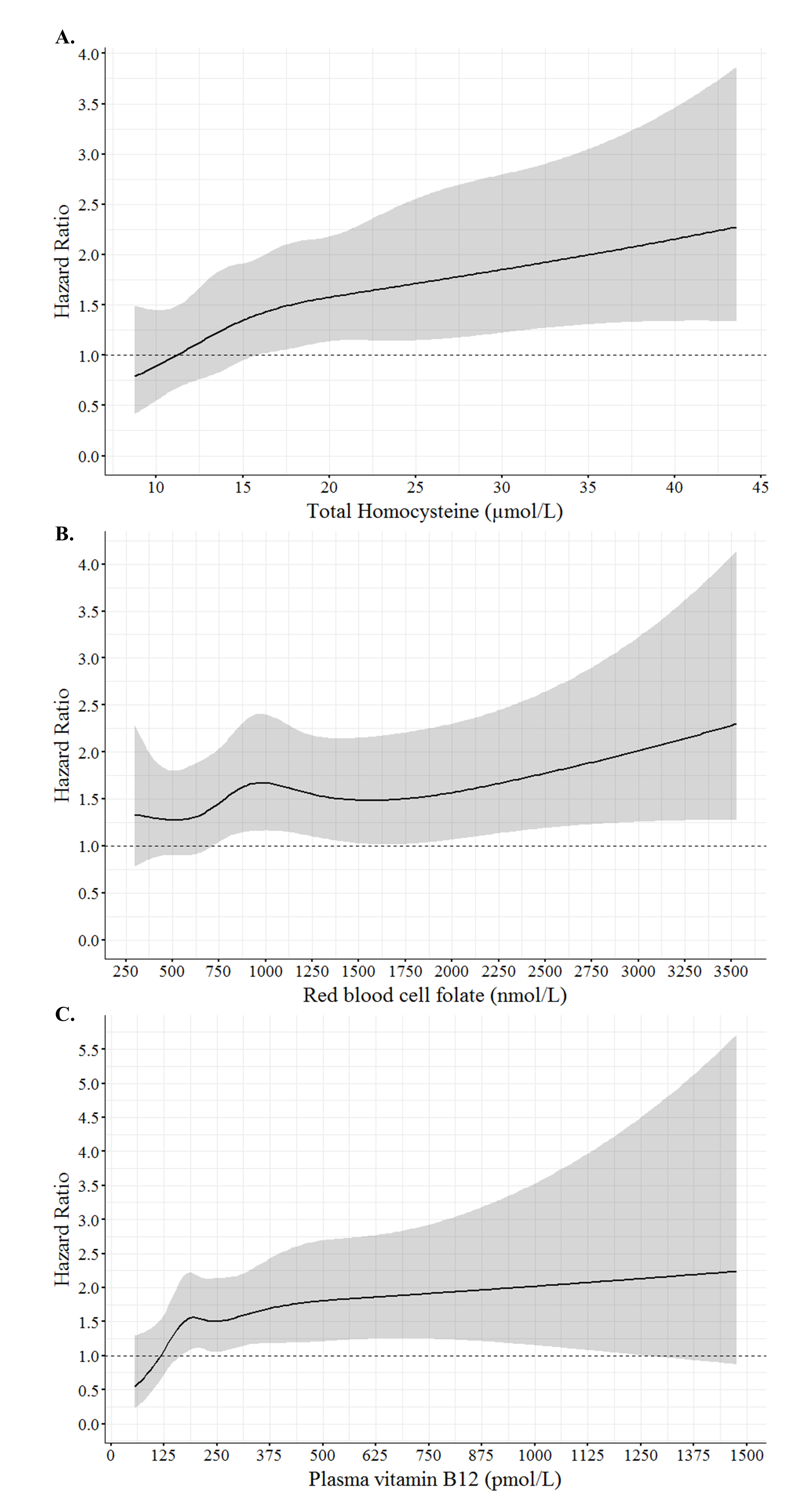

Supplement: Supplemental Figure 3 [file gly035_suppl_supplemental_figure_3.png]
